# A bibliometric analysis of building information modelling implementation barriers in the developing world using an interpretive structural modelling approach

**DOI:** 10.1016/j.heliyon.2023.e18601

**Published:** 2023-07-25

**Authors:** Georgina Esi Takyi-Annan, Hong Zhang

**Affiliations:** School of Architecture, Southeast University, No.90 Chengxian Street, Xuanwu District, Nanjing, Jiangsu Province, 210096, China

**Keywords:** Building information modelling, Developing countries, Bibliometric analysis, Interpretive structural modelling

## Abstract

Over the past 20 years, the phrase “Building Information Modelling” (BIM) has spread throughout the Architecture, Engineering and Construction (AEC) industries. BIM usage in the construction industry is vital in the revolution towards Industry 4.0 in the AEC Industry. BIM contributes to this change due to its automatization and sustainability features. However, there are growing concerns about its implementation in the developing world context. The BIM Implementation Barriers (BIMIBs) in individual countries and on a global scale have been examined in a variety of studies and works of literature, but two research questions are still open; (1) what specific BIMIBs are the AEC industries in the developing world encountering the most, and (2) what is the interrelationship between these barriers? Through a combination of expert interviews and a bibliometric analysis of published relevant empirical studies on the subject, the aim of this study is to identify these frequently occurring BIMIBs in the developing world and to determine the interrelationships between these barriers using an Interpretive Structural Modelling (ISM) approach and MICMAC analysis. The study identified the 14 BIMIBs with ‘high associated cost’ as the most fundamental of all. A comparison of the study's findings and a proposed 3-level barrier mitigation strategy with other studies identified the lack of governmental support for BIM implementation and research as a root cause of majority of the BIMIBs identified in the developing world. This study lays forth the knowledge base for future studies in the area of BIM implementation in the developing world.

## Highlights

• Bibliometric analysis was performed on 124 relevant empirical studies using the visualization of similarity software.
• The ISM approach and MICMAC analysis determined the interrelationships between 14 BIM implementation barriers in the developing world.
• TThere was no active collaboration between authors of the subject under discussion, a reflection of the industry's anti-collaborative culture
• ‘High associated cost’ was the most significant and fundamental barrier that affects BIM implementation in the developing countries..

## 1 Introduction

Traditionally, 3D drawings and paper-based documentations were used to design, erect, and maintain infrastructure and buildings. These are increasingly being replaced by 3D computer-aided designs (CAD), which are now being overtaken by Building Information Modelling (BIM) [1]. BIM as a technology or tool was developed over one and a half decades ago [2] and has become a very critical instrument in the Architecture, Engineering and Construction (AEC) industries for innovation and productivity. Currently, the BIM model is employed in domains such as construction management, cost control, planning, sustainability, and safety education, in addition to 3D modelling [2]. The construction industry is one of the least sustainable industries in the world because about a half of all the non-renewable resources consumed by humans are used in construction [3]. In addition, buildings contribute roughly 20% to city air pollution, 50% to climate change gases, 40% to drinking water pollution, 50% to landfill garbage, and 50% to ozone depletion globally [3].

Integrating BIM into building construction has numerous advantages over the course of a project's lifecycle. Some of these advantages in the building construction business include the following; (1) an improved communication and coordination, (2) an increased work speed and accuracy, (3) visualization and walkthrough abilities, (4) high-quality documentation, (5) improved project performance and quality, (6) ability to decrease waste and lower construction costs, (7)budget control and lean management abilities, and (8) ability to create new income and business opportunities [4,5].

While many construction projects in the developed world context are gradually implementing BIM, its implementation in the developing world is hampered by numerous challenges [2]. Recently, BIM research has increased dramatically on a global scale [6,7]. In order to determine the development trend, various research investigations have attempted to build an overall review of the growing body of BIM papers utilizing manual review, scientometric review, bibliometric review, or latent semantic review [8]. Despite the fact that the adoption of BIM varies among businesses, nations, and continents, these existing studies frequently take a global perspective of the evolution [8]. This strategy is viewed as being unrepresentative of BIM development in various nations and continents who are at the early stages of BIM implementation [8]. There are also numerous empirical studies on the BIM Implementation Barriers (BIMIBs), in specific developing nations [[9], [10], [11], [12]] however, there is a considerable amount of neglect in this area. It is crucial to remember that the various barriers do not exist in isolation, rather, they form intricate correlations that prevent successful BIM implementation [13]. No study has examined all of these distinct empirical studies to determine interrelationships between the most prioritized and most occurring BIM implementation barriers in the developing world.

Through a bibliometric evaluation of published relevant empirical studies on the subject, the aim of this research is to identify these frequently occurring BIM implementation barriers in the developing world and to determine the interrelationships between these barriers using an Interpretive Structural Modelling (ISM) approach and MICMAC analysis. The study would raise awareness of the BIM software in the developing world, propose workable solutions to address these barriers, to enhance practitioners' trust in BIM usage and to serve as a solid platform for future research in the field.

## 2 Materials and methods

There are three primary types of literature reviews: systematic review, meta-analysis, and Bibliometric Analysis. The study adopts the use of the bibliometric analysis method to review related literature due to the large amount of data to be studied. A bibliometric analysis is a method for discovering and analyzing massive amounts of data and can be used to spot developing patterns, research components, and patterns of collaboration on a given subject [14]. Managing such data can be done using other review techniques such as meta-analysis. Although meta-analysis and bibliometric analysis are both quantitative review techniques, in a bibliometric analysis, the data to be examined should not all be uniform. In addition, the bibliometric analysis provides information on the quantitative evaluation of the elements within an article such as keywords, publications, publishers, and citations. The bibliometric analysis is preferable for this study because this method highlights the most recent developments in the study, research direction and trends.

The data for the bibliometric study was obtained from the Web of Science (WoS) database. WoS was chosen because it has proven to be a reliable source of data and information over time, as the world's oldest, most widely used, and authoritative data base of research publications, with over 155 million records, including journals, books, and proceedings, as well as over 70 million patents [15]. In order to be able to use published work with confidence and to control quality and eliminate flaws, only peer reviewed articles based on empirical studies were collected. Empirical study methods are employed largely in quantitative research and often involve the systematic collection and analysis of data based on observation and evidence [16]. Only published studies based on data collected from the industry through methods such as questionnaire survey, delphi methods, focus group discussions, case studies, and interviews were considered.

Even though the exceptions involved in the data collection process such as the exclusion of review studies and the focus on only BIMIBs in the developing countries were expected to greatly reduce the amount of the final relevant papers for this study, the search process on WoS was very laborious. A minimum of 152 searches were made using the keywords ‘building information modelling’ + ‘name of a developing country’. For instance, if the country in question is Ghana, the keywords for the search on WoS would be ‘Building information modelling Ghana’. The ‘quick filter search’ command on WoS was then used to exclude the inclusion of review papers. This was done for each of the 152 developing countries identified based on IMF's classification of countries (find link here: List of 152 developing countries (worlddata.info)). After, the results were sorted out for each country through a perusal of their abstracts, methodologies and results to identify the studies that addressed the BIMIBs in the respective countries using empirical data. This was done over a three-month period (July, August and September 2022). A total of 124 relevant studies were eventually collected and used for this study.

**Fig. 1:**
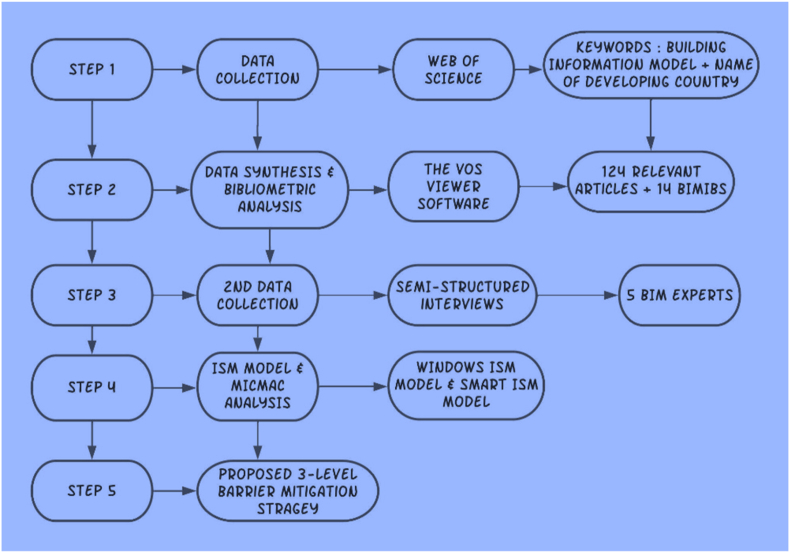
Research methodology.

Lastly, in order to establish the interrelationships between the BIMIBs, the study adopted the use of experts’ interviews, MICMAC analysis and the ISM model. Steps taken in this process would be explained further in details in section 3.3. Fig. 1 summarizes the methodology used.

## 3 Results and discussions

This section would present the findings and analysis of the study. The following are the labels for the 124 studies reviewed in this paper;

R1 [17], R2 [18], R3 [19], R4 [10], R5 [20], R6 [21], R7 [22], R8 [23], R9 [24], R10 [25], R11 [26], R12 [13], R13 [27], R14 [28], R15 [29], R16 [30], R17 [31], R18 [32], R19 [26], R20 [33], R21 [34], R22 [35], R23 [36], R24 [37], R25 [38], R26 [39], R27 [40], R28 [41], R29 [42], R30 [43], R31 [44], R32 [45], R33 [46], R34 [47], R35 [48], R36 [49], R37 [50], R38 [51], R39 [52], R40 [53], R41 [54], R42 [55], R43 [56], R44 [57], 45 [58], 46 [59], R47 [60], R48 [61], R49 [62], R50 [63], R51 [64], R52 [65], R53 [66], R54 [67], R55 [68], R56 [69], R57 [70], R58 [71], R59 [72], R60 [73], R61 [74], R62 [75], R63 [76], R64 [77], R65 [78], R66 [79], R67 [80], R68 [81], R69 [82], R70 [83], R71 [84], R72 [85], R73 [86], R74 [87], R75 [88], R76 [89], R77 [90], R78 [91], R79 [92], R80 [93], R81 [94], R82 [95], R83 [96], R84 [97], R85 [98], R86 [99], R87 [100], R88 [101], R89 [102], R90 [103], R91 [104], R92 [105], R93 [106], R94 [107], R95 [108], R96 [109], R97 [110], R98 [111], R99 [112], R100 [113], R101 [114], R102 [115], R103 [116], R104 [117], R105 [118], R106 [118], R107 [119], R108 [120], R109 [121], R110 [122], R111 [123], R112 [124], R114 [125], R115 [12], R116 [126], R117 [127], R118 [128], R119 [129], R120 [130], R121 [131], R122 [132,133], R123 [133], R124 [134].

### 3.1 Data synthesis and analysis

In this section, the data collected from the 124 empirical studies on BIM implementation barriers are compiled and illustrated using visualization techniques such as graphs, tables, charts, etc. Fig. 1 displays the number of empirical documents released on BIMIBs in developing countries annually.

**Fig. 2:**
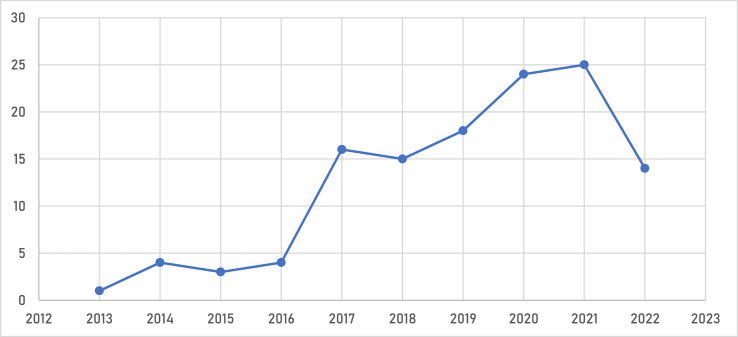
The number of articles based on empirical studies released on BIMIBs in developing countries annually.

According to Fig. 2, the study observed two major eras between 2013 and 2022. With an average of 3 papers per year in Period I (from 2013 to 2016), the number of empirical studies on BIMIBs in developing countries shows an unstable growing tendency. The growth rate was fluctuating. There were additions in the years 2014 and 2016 whereas in 2013 and 2015 there were reductions in the number of publications per year. Period I can be considered a transitional phase in which, despite the fact that there was a general increase in interest in BIM research, the growth in scientific production was not stable even though the BIM concept dates back to the 1970s and BIM dissemination research in developing countries first began in 2006 [7]. Between 2006 and 2013, there was no empirical studies on BIMIB because within this era, BIM research in developing nations in general was in its early stages and researchers were still looking for a foothold [7]. However, research on BIMIBs in the developing countries experienced a very remarkable uptick in 2017. This is because in 2017, BIM research generally saw a surge in publications with a greater emphasis on sustainability, life cycle evaluations, interoperability, and BIM education [6]. This was followed by a consistent and noticeable rise in Period II (from 2018 to 2022), with an average of 19 documents produced annually, an increase of 533% from Period I's average. The most fruitful year thus far in empirical studies on BIMIBs in develping countries is 2021 with a total of 25 five papers. With this recent continuous increase, it is clear that BIM awareness is steadily growing in developing nations.

**Fig. 3:**
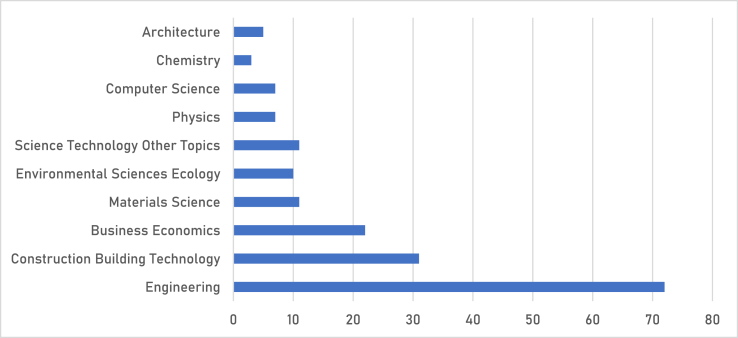
Research areas of journals publishing empirical studies on BIM implementation barriers.

The top ten (10) journals that publish empirical studies on BIMIBs in developing countries are shown in Fig. 3. The majority of the journals lie primarily under Engineering followed by Construction and Building Technology. Also prominent among them are the journals that are not directly related to the field of Architecture, Engineering and Construction (AEC) such as Business Economics, Physics and Chemistry. This is excellent in that a wider audience is being reached for raising awareness on the subject at hand.

**Fig. 4:**
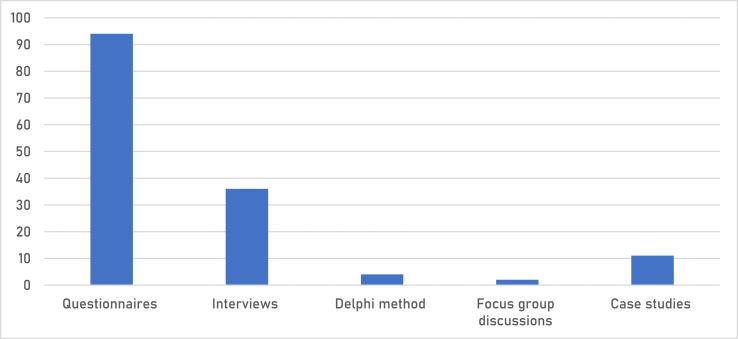
Data collection methods.

The data collection methods adopted by all 124 articles have been summarized in Fig. 4. They include the delphi method, focus group discussions and interviews, case studies, and surveys (questionnaires and interviews). Four (4) studies, which accounts for 3% of the total adopted the use of the Delphi method. Eight (8) studies) accounting for 3% of the total used case studies as a data collection method. Twenty-nine (29) percent of the studies used interviews whereas only two (2) percent used focus group discussions. Majority of the studies used questionnaires (Seventy-five percent (75%) of the studies (95 studies) used questionnaires). Questionnaires, as the most extensively used survey tool in the social sciences, when well administered give a wide range of information, from reporting participant demographics and backgrounds to recording opinions, reporting factual knowledge, assessing psychometric qualities, and so on [135]. The sample spaces of the studies that used questionnaires were not taken into consideration. According to research [135], how large or small a sample space is not as important as its ability to give a desirable accuracy and confidence level in that data collected. The response rates were also not taken into consideration. No response rate, no matter how high or low, is a guarantee of greater or lesser data accuracy [136]. Table 1 summarizes the results [136].

**Table 1:** Data collection methods.

Method	References	T	%
Questionnaires	R1 R2 R4 R5 R6 R7 R8 R9 R10 R11 R12 R13 R15 R17 R19 R20 R21 R23 R24 R26 R27 R28 R29 R30 R31 R32 R33 R34 R35 R37 R38 R40 R41 R42 R43 R44 R45 R46 R48 R49 R50 R51 R52 R53 R54 R56 R57 R60 R63 R64 R66 R68 R69 R70 R71 R72 R74 R75 R78 R79 R80 R81 R82 R83 R84 R86 R88 R89 R90 R91 R92 R93 R94 R95 R96 R97 R99 R100 R101 R102 R103 R106 R109 R110 R111 R112 R113 R114 R115 R116 R117 R118 R120 R123	94	75
Interviews	R3 R12 R13 R18 R24 R25 R30 R36 R39 R44 R45 R47 R48 R49 R53 R55 R58 R60 R62 R67 R68 R76 R77 R79 R81 R85 R87 R90 R92 R98 R99 R105 R106 R107 R108 R113	36	29
Delphi method	R14 R16 R67 R94	4	3
Focus group discussions	R35 R65	2	2
Case studies	R2 R59 R61 R62 R68 R77 R93 R105 R121 R122 R124	8	9

When it comes to BIM adoption, the importance of stakeholders in the construction sector cannot be underestimated. Around the world, different stakeholders play diverse roles in BIM implementation and use BIM for various goals. Architects utilize BIM to evaluate and develop project designs, whilst contractors use it to manage and schedule BIM activities. In order to fully understand the BIM implementation challenges in the developed world, there is the need to involve these stakeholders in the study. More than 10 stakeholders were identified in this study. They include architects, engineers, project managers, interior designers, clients and building owners (see Table 2). Architects are typically the lead designers on most construction projects, and they are in charge of creating conceptual ideas, detailed designs, construction-level information, and design analysis [137]. Architects made up the greatest percentage of stakeholders who participated in the investigations, accounting for roughly fifty-one (51) percent, followed by contractors and engineers, who accounted for forty-four (44) and forty-one (41) percent of the total number of studies respectively. This makes Architects, engineers and contractors the top 3 stakeholders in empirical studies on BIMIBs in the developing world.

**Table 2:** Stakeholders.

Stakeholders	References	T
Architects/Designers	R1 R2 R4 R5 R6 R7 R10 R11 R12 R13 R15 R17 R23 R26 R32 R35 R38 R42 R43 R44 R44 R45 R47 R48 R50 R54 R56 R57 R60 R62 R63 R64 R66 R67 R68 R69 R71 R72 R73 R74 R75 R76 R79 R80 R81 R82 R83 R88 R90 R92 R93 R95 R96 R97 R100 R101 R102 R107 R108 R112 R114 R118 R120 R121	64
Contractors	R1 R9 R11 R13 R16 R19 R20 R21 R23 R24 R26 R27 R28 R31 R33 R34 R36 R39 R41 R43 R44 R45 R47 R48 R49 R50 R58 R63 R64 R66 R70 R71 R74 R75 R79 R80 R81 R83 R86 R92 R93 R96 R97 R100 R101 R102 R104 R106 R109 R112 R114 R116 R118 R120	54
Builders/Constructors	R4 R7 R17 R35 R50 R90 R95 R116	8
Quantity Surveyors	R5 R6 R7 R10 R25 R26 R27 R29 R34 R47 R51 R56 R57 R71 R83 R90 R114	17
Engineers	R1 R4 R5 R7 R10 R12 R15 R16 R18 R26 R27 R32 R35 R36 R38 R44 R45 R48 R50 R51 R54 R56 R57 R60 R62 R64 R65 R66 R67 R69 R76 R80 R81 R82 R83 R88 R92 R93 R95 R96 R97 R100 R101 R104 R105 R106 R109 R111 R112 R114 R118 R120	52
BIM Experts/managers	R3 R14 R20 R35 R36 R45 R56 R62 R77 R87 R97 R104 R105 R108	15
Clients/Owners	R4 R9 R13 R16 R17 R16 R20 R21 R23 R24 R27 R31 R36 R38 R43 R44 R45 R48 R49 R51 R60 R66 R71 R79 R80 R90 R93 R100 R101 R102 R106 R109 R112 R116	34
Land/Estate/Valuation surveyors	R5 R6 R7 R90	3
Managers	R27 R47 R51 R55 R56 R80 R81 R105 R106	9
Facility managers	R6 R12 R28 R43 R44 R45 R57 R79 R84 R92 R107 R116	12
Consultants	R9 R11 R17 R19 R21 R22 R24 R28 R31 R34 R36 R38 R49 R50 R54 R64 R65 R70 R71 R74 R95 R96 R104 R106 R109 R112 R114 R116	28
Project Managers	R5 R10 R15 R17 R36 R50 R51 R54 R55 R60 R63 R65 R66 R71 R81 R83 R93 R96 R100 R101 R105 R106 R116 R118	24
Academicians	R8 R11 R12 R15 R17 R27 R28 R30 R32 R35 R50 R58 R63 R65 R79 R80 R81 R91 R103 R110 R112 R114 R115 R122	24
Government agencies	R16 R17 R32 R35 R43 R44 R48 R70 R74 R79 R97 R116	12
Interior designers	R37 R40	2
Manufacturers, suppliers	R34 R44 R45 R100 R101 R108	6
Others e.g. Supervisors, planners, products managers, experts	R18 R23 R47 R52 R56 R62 R78 R81 R85 R89 R92 R93 R94 R96 R98 R99 R100 R101 R107 R111 R112 R113 R123	23

**Fig. 5:**
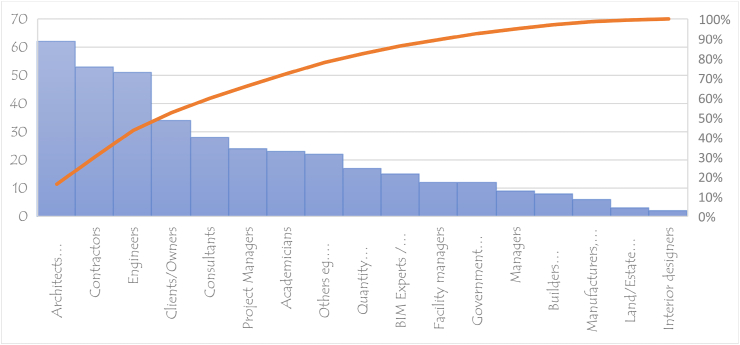
Majors Stakeholders in research on BIMIB in developing countries.

Clients were regarded as the fourth-largest stakeholder in this study and this is excellent because they are crucial to the deployment of BIM (see Fig. 5). Client organizations are widely recognized as crucial actors in advancing construction innovation in order to overcome BIM barriers, but the specifics of the client role are less obvious, and it is debatable whether BIM innovation should primarily be client-led or not [138]. Research on BIM implementation supports the idea of an involved client who actively participates, demands the technology in procurement, and generally influences its adoption [138].

### 3.2 Bibliometric analysis

Bibliometric analysis is a beneficial tool for gaining information of the advancements and connections among the study topics in various disciplines [139]. The different aspects of bibliometric review adopted by this study are the co-authorship networks, co-authorship of countries, citation of countries and the cooccurrence of keywords.

#### 3.2.1 Co-authorship network

**Fig. 6:**
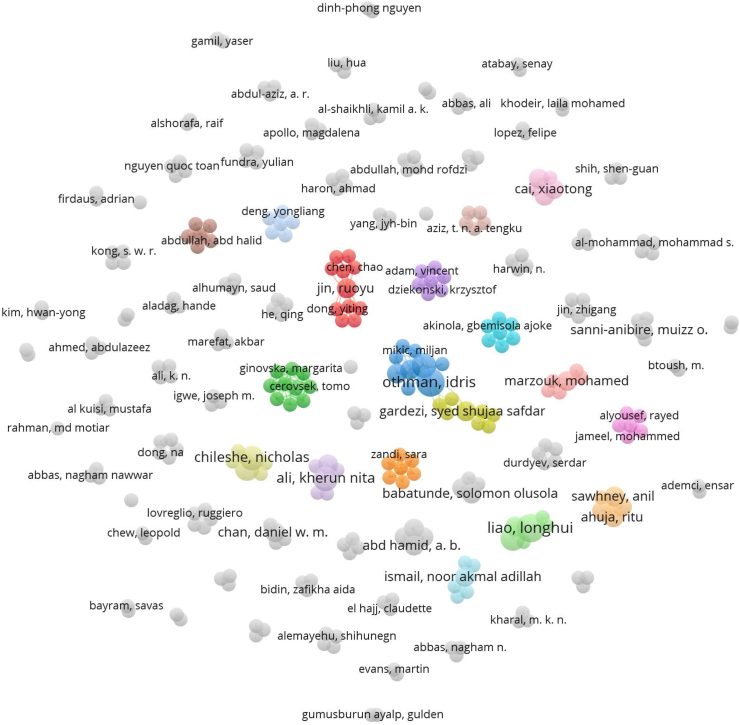
Network visualization of co-authorship network.

Scientific collaboration networks are a defining feature of modern academic research and researchers are now members of teams that combine complementary abilities and diversified perspectives to work toward shared objectives rather than acting as individual players [140]. The co-authorship network is a display of the relationship between two (2) or more authors of a subject area. By identifying players and their relationships, the co-authorship network analysis illuminates the social structure of the networks [140]. As shown in Fig. 6, a network of authors who have made major contributions to BIMIB research and trends in the developing countries has been formed.

The paper utilized the VOS viewer software's analytical techniques. 391 authors in all, including both lead authors and contributing authors, contributed to the 124 studies that were reviewed. The threshold for the minimum number of documents of an author as well as that of the minimum number of citations per author was set at 1.317 out of the 391 authors of the 124 documents met the relevant criteria. For each of the 317 authors, the total strength of the co authorship links with other authors were calculated and the authors with the greatest total link strength were selected. The top 15 prolific authors who have made the most significant scientific and intellectual contributions in the field of empirical studies on BIMIBs in developing countries are shown in Table 3.

**Table 3:** Top 15 prolific authors who have made the most significant scientific and intellectual contributions in the field of empirical studies on BIMIBs in developing countries.

Author	Documents	Citation	Total link strength
Othman, Idris	4	100	16
Al-Ashmori, Yasser Yahya	3	92	12
Arman, Y·H. Mugahed	2	90	10
Jin, Ruoyu	2	90	10
Rahmawati, Yani	2	90	10
Cerovsek, Tomo	1	3	9
Funtik, Tomas	1	3	9
Ginovska, Margarita	1	3	9
Ivanov, Risto	1	3	9
Krleski, aleksandar	1	3	9
Panchevski, Igor	1	3	9
Perez Arnal, Ignasi	1	3	9
Sandeva, Ivana	1	3	9
Spasevska, Hristina	1	3	9
Stojanovska-Georgievska, Lihnida	1	3	9

Based on the number of publications, citation scores and link strength, the research impact was determined [6]. A perusal of Table 3 reveals that Othman, Idris (4 documents, 100 citations), Al-Ashmori, Yasser Yahya (3 documents, 92 citations), Amran, Y.H. Mugahed (2 documents, 90 citations), Jin, Ruoyu (2 documents, 90 citations) have incredibly substantial scientific impacts in the subject area under consideration. Chan, Daniel W.M (2,152) is also very well regarded in the field, particularly given the number of citations.

**Fig. 7:**
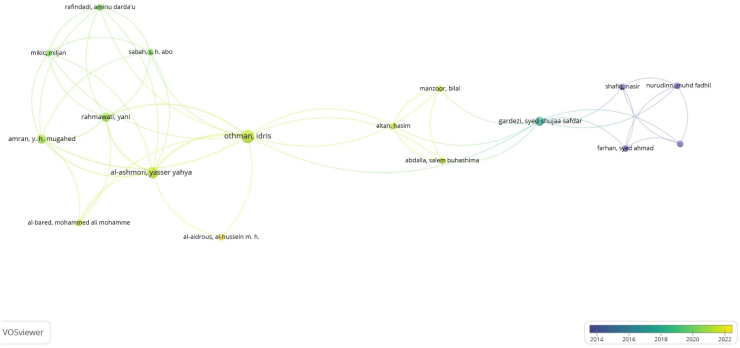
Overlay visualization of 17 linked authors.

With 317 items from the network, 85 clusters of co-authorship were found, as shown in Fig. 6. A total of 523 links were formed between the authors with a total link strength of 553. It is important to note that, most of the 317 items (authors) in the network are not linked together. This demonstrates a general lack of collaboration between the authors. The largest set of connected items consists of 17 items. This has been illustrated by Fig. 7. The clusters formed demonstrate an active research collaboration among these few scholars particularly in cluster 3 (labelled in blue), with authors like Othman, Idris, Al-Ashmori, Yasser Yahya. Authors labelled in yellow such as Al-Aidrous, Al-Husein are the latest researchers on BIMIBs in the developing countries.

#### 3.2.2 Citation of countries

**Fig. 8:**
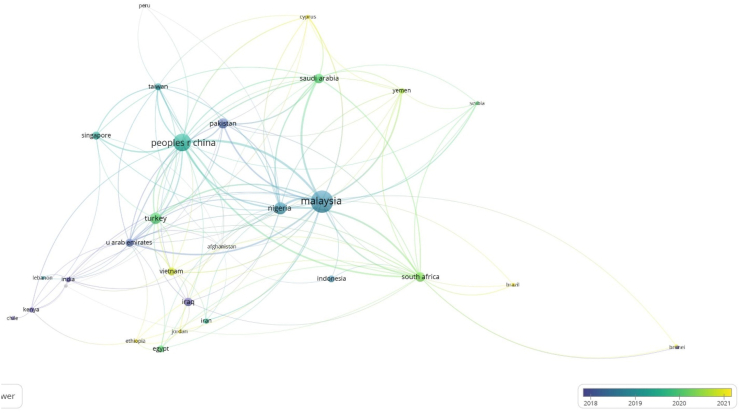
Overlay visualization of the citation network of countries.

To acquire information into the state of empirical studies on BIMIBs in developing countries, a citation network of countries was created. The final network as shown in Fig. 8 contains 6 clusters, 130 linkages, and a total link strength of 352. The weight of the items determines the sizes of the nodes, hence, for the purpose of this study, the sizes of the nodes in the network indicate the quantity of publications from the individual countries. The links connecting these countries show how many co-authorship relationships there are between these countries.

**Fig. 9:**
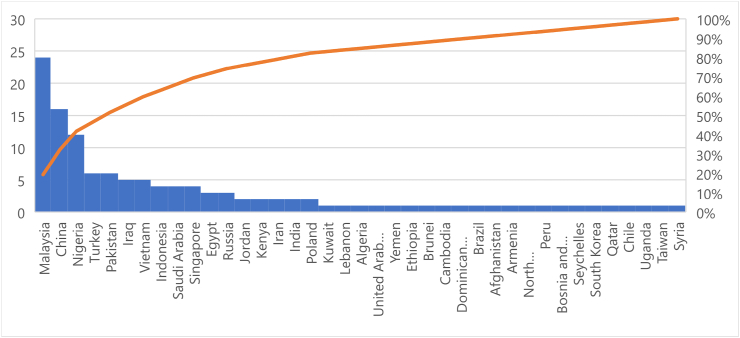
Number of publications per country.

The color bar in Fig. 8's lower right corner serves as the basis for the trend and it is represented by the color of the link. A quick glance at the color bar reveals that Malaysia and the People's Republic of China have more publications than the rest of the countriesm, most of which were published in 2019. By implication, countries like Malaysia and the People's Republic of China who have a large body of work have moved further in BIMIBs research than other emerging nations. This could be an indication that among the developing countries, the Asian countries attach a lot of value to BIM development and research. This assumption is supported by a study carried out on global BIM research in the AEC industry [141]. As a result, they can be regarded as leaders of research on BIMIBs in the developing world. Countries such as Vietnam, Ethiopia, Jordan, Brunei and Brazil are indicated with yellow nodes which reflect the fact that research on BIMIBs in these countries is more recent. However, BIM implementation in these countries are still at their infancy stages hence the fewer number of recorded publications [7]. Based on the previous assumption, Nigeria ranks number 1 among the African countries in BIM implementation and number 3 among the developing countries (see Fig. 9).

**Fig. 10:**
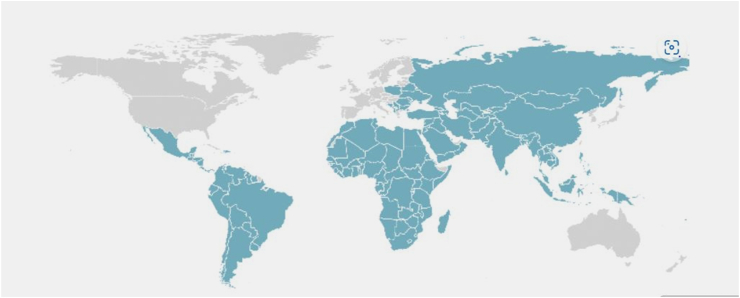
The distribution of the developing countries around the world.

**Fig. 11:**
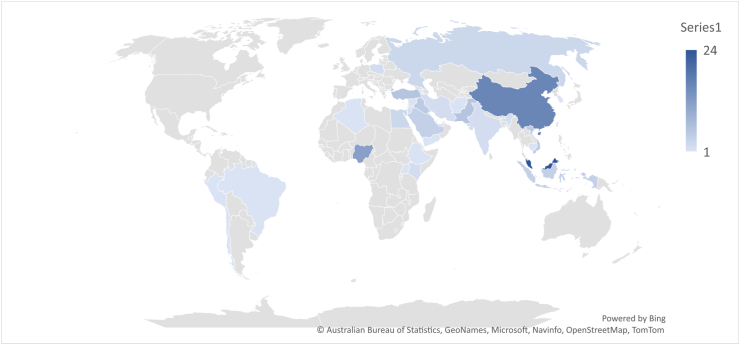
Distribution of the developing countries on which empirical studies have been carried out on the BIMIBs.

Fig. 10 illustrates the portion of the world covered by developing countries whereas Fig. 11 covers the portion of the developing countries that have recorded published research in the area of BIMIBs. Majority of the developing countries are located at South America, Africa (the whole of Africa) and Asia. There is a vast amount of ground to be covered in Africa, an indication of BIM implementation at an infancy stage in Africa. The research highly recommends that more research on the study area should be done in Africa, so that assumptions made in the future can be supported by data rather than by a dearth of literature. Table 4 summaries data collected regarding the countries and their references.

**Table 4:** Developing countries that have conducted empirical studies on the BIMIBs.

Countries	Reference	N
Algeria	R1	1
Egypt	R2 R3 R99	3
Ethiopia	R4	1
Uganda	R5	1
Nigeria	R6 R7 R8 R9 R84 R89 R90 R91 R114 R115 R116 R118	12
Kenya	R10 R86	2
China	R11 R12 R13 R14 R15 R16 R17 R19 R20 R85 R87 R100 R101 R102 R112 R113	16
Malaysia	R18 R25 R26 R27 R28 R29 R30 R31 R32 R33 R34 R35 R36 R37 R38 R39 R40 R41 R103 R106 R107 R108 R109 R110	24
Indonesia	R21 R22 R23 R24	4
Taiwan	R42	1
Singapore	R43 R44 R45 R92	4
Vietnam	R46 R47 R48 R93 R105	5
Iran	R49 R94	2
Pakistan	R50 R51 R52 R68 R95 R117	6
Iraq	R53 R54 R55 R56 R96	5
Jordan	R57 R58	2
Kuwait	R59	1
Lebanon	R60	1
Saudi Arabia	R61 R62 R63 R64 R104	4
Turkey	R65 R66 R67 R69 R88 R98 R104	6
United Arab Emirates	R70	1
Yemen	R71	1
India	R72 R73	2
Brunei	R74	1
Cambodia	R75	1
Dominican Republican	R76	1
Brazil	R77	1
Afghanistan	R78	1
Armenia	R79	1
North Macedonia	R80	1
Peru	R81	1
Bosnia and Herzegovina	R82	1
Seychelles	R83	1
South Korea	R97	1
Qatar	R104	1
Syria	R111	1
Russia	R119, R1123, R24	3
Chile	R120	1
Poland	R121, R122	2
Total		124

#### 3.2.3 Top productive and influential countries of authors of empirical studies on BIM implementation barriers

**Fig. 12:**
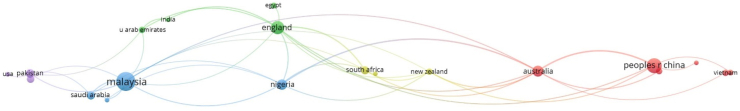
Network visualization of top 20 countries with the most authors and author collaborations.

The 391 authors identified in section 3.2.1 by are from 50 different countries. With a total of 32 articles, Malaysia ranks as the number 1 country with authors producing empirical studies on BIMIBs in the developing world, followed by the People's Republic of China with a total of 20 articles and England ranking number 3 with 11 articles. With regard to total link strengths, Australia (11 documents, 227 citations) ranks number 1 with total link strength of 22. England (15 documents, 258 citations) and Malaysia (32 documents, 339 citations) rank 2nd and 3rd with total link strengths of 21 and 18 respectively. Nigeria (10 documents, 145 citations) and the People's Republic of China (20 documents, 522 citations) rank 4th and 5th with total link strengths of 16 and 15 respectively (see Table 5). China tops as the leading county with authors whose articles have been cited the most in this domain of discussion. Fig. 12 shows the top 20 leading countries with the most authors and author collaborations in research on BIMIBs in the developing world.

**Table 5:** Top 5 countries producing authors on the subject in question.

Author	Documents	Citation	Total link strength
Australia	11	208	22
England	15	235	21
Malaysia	35	324	18
Nigeria	10	122	16
Peoples Republic of China	22	485	16

Table 5 Top 5 productive and influential countries of authors of empirical studies on BIM Implementation challenges.

As illustrated by Fig. 12 and Table 5, some of the top 20 countries producing authors in the study domain include developed countries such as South Korea, New Zealand, United States of America and the Netherlands with the top 2 (with regards to total link strength) being England and Australia. This demonstrates that these developed countries have a strong research strength and influence in BIM implementation and research in the developing countries.

**Fig. 13:**
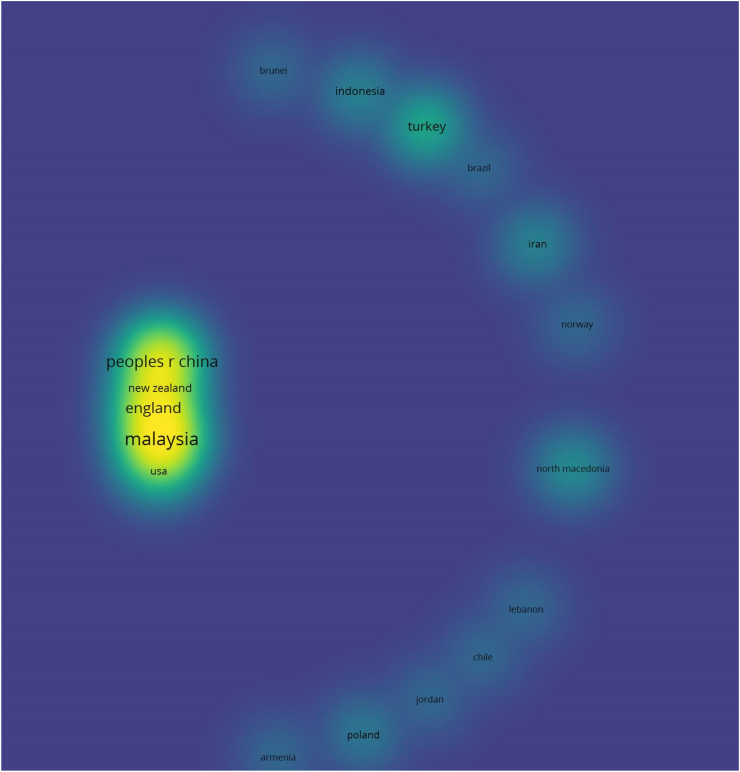
Density visualization of co-authorship of countries network.

When the thresholds for minimum number of authors and minimum number of citations are set at 1 each, 42 out 50 countries meet the criteria. According to this network as illustrated in Fig. 13, there are not many joint research projects on BIMIBs in the developing countries. The large number of lone authors without links make this clear. It is safe to state that BIM research at large is conducted in isolation in the developing countries. This could be a reflection of the industry's anti-collaborative culture [7]. Effective BIM dissemination research in developing nations would require a shift in the prevalent culture among researchers.

#### 3.2.4 Keywords co-occurrence and cluster identification

**Fig. 14:**
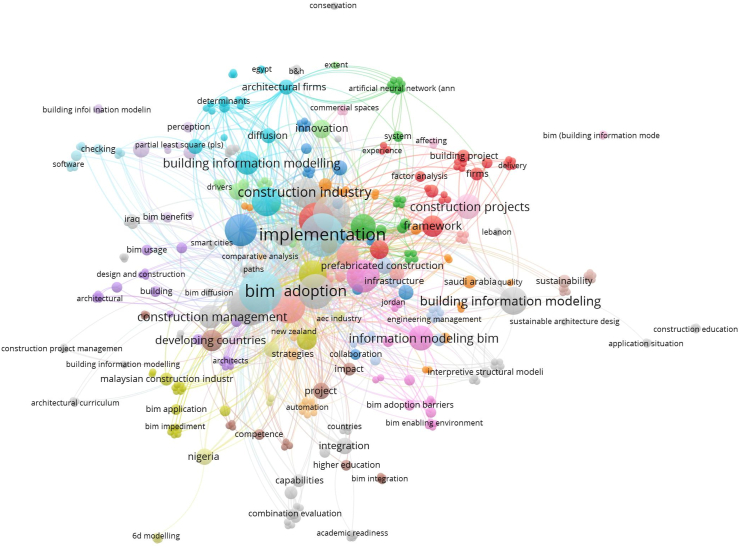
Network visualization of co-occurrence of keywords.

The analysis of keywords aids in identifying important study subjects within the subject matter. The term “co-occurrence” describes the proximity or shared presence of related keywords [6]. It is a network of keywords that frequently appear together in papers which correspond to the themes of research publications [8]. Similar keywords based on the same topic but with different meanings may also be included in co-occurrence, and the closeness of keywords has a direct bearing on the degree of co-occurrence [139]. Using the VOS viewer software, a co-occurrence network was created from the complete volume of keywords. A total of 389 keywords were discovered as shown in Fig. 14.

The top 10 reoccurring words (see Table 6) in all of the 118 articles collected are (1)Implementation, management, (2)BIM, (3)adoption, (4)barriers, (5)Building Information Modelling (6), Building Information Modelling (7), (8)Construction, (9)challenges and (10)benefits. Keywords 6 and 7 were used interchangeably by authors. It is however not considered a problem because “modelling” is simply the American spelling of “modelling”, hence, there is no change in meaning when authors use them interchangeably.

**Table 6:** Top 10 keywords.

Keywords	Occurrences	Total link strength
1. Implementation	32	297
2. Management	22	250
3. BIM	32	217
4. Adoption	22	214
5. Barriers	20	211
6. Building Information Modelling	24	198
7. Building information Modelling	27	193
8. Construction	19	174
9. Challenges	18	152
10. Benefits	14	151

**Fig. 15:**
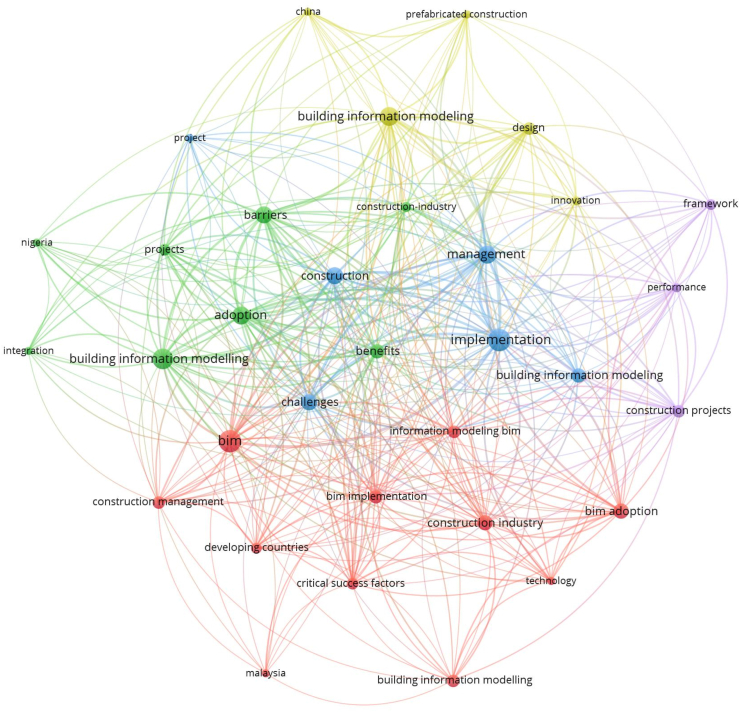
Co-occurrence of top 31 keywords.

For clarity and in order to reduce the number of key words to only important ones, the threshold for the minimum number of occurrences of a key word was set at 5. Only 33 out of the 389 key words met this requirement. In all, a total of five clusters were found with three hundred and seventy-nine (379) links with a total link strength of 951 as illustrated by Fig. 15. The keywords in these five clusters would be discussed.

Cluster #1 has a red label. This cluster has about 11 items. Keywords that can be associated with this cluster include ‘developing countries’, ‘BIM’, ‘BIM implementation’, ‘Malaysia’, ‘technology’, ‘critical success factors’, ‘BIM adoption’, ‘technology’ and ‘construction industry’. ‘BIM’ as a keyword has the strongest links in this cluster with 31 links, a total link strength of 81 and 32 occurrences. Cluster #2 has a green label. This cluster has about 7 items. Keywords that can be associated with this cluster include ‘barriers’, ‘Nigeria’, ‘Building Information Modelling’, ‘benefits’, ‘adoption’ and ‘projects’. ‘Barriers’ as a keyword has the strongest links in this cluster with 29 links, a total link strength of 110 and 20 occurrences. Cluster #3 has a blue label. This cluster has about 5 items. Keywords that can be associated with this cluster include ‘Building Information Modelling’, ‘implementation’, ‘management’‘, construction’ and ‘challenges’. ‘Implementation’ as a keyword has the strongest links in this cluster with 30 links, a total link strength of 134 and 33 occurrences. Cluster #4 has a yellow label. This cluster has about 5 items. Keywords that can be associated with this cluster include ‘design’, ‘Building Implementation Modelling’, ‘prefabricated construction’, ‘China’ and ‘innovation’. ‘Building Information Modelling’ as a keyword has the strongest links in this cluster with 26 links, a total link strength of 92 and 24 occurrences. Cluster #5 has a purple label. This cluster has only 3 items. Keywords that can be associated with this cluster include ‘construction projects’, ‘performance’ and ‘framework’. ‘Construction projects’ as a keyword has the strongest links in this cluster with 23 links, a total link strength of 55 and 11 occurrences.

The general observations from these clusters are as follows; (1) BIM or Building Information modelling/modelling appeared in all 5 of the clusters which emphasizes that the data collected is just right for this paper because that is what the whole of this paper is about. (2) Names of countries that appeared in the clusters are a true reflection of the developing countries with the most published empirical data on the BIMIBs. The countries are Malaysia (cluster 1), China (cluster 4) and Nigeria (cluster 2). (3) The top key words in each of the 5 are BIM (cluster 1), barriers (cluster 2), building information modelling (cluster 3), implementation (cluster 4), construction industry (cluster 5). These keywords altogether give a vivid idea on the whole aim or essence of this paper which has to do with assessing the interrelationships between BIM Implementation Barriers in the Architecture, engineering and Construction industries of the developing world.

### 3.3 Interpretive structural modelling (ISM) and MICMAC analysis

**Fig. 16:**
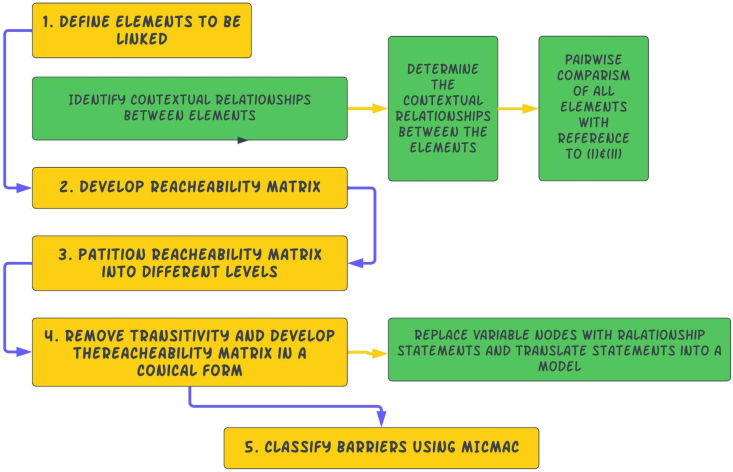
The ISM methodology process adopted by the study.

The investigation of the interrelationships between the elements in a complex system employs a variety of methodologies. The Analytical Network Process (ANP) and Interpretive Structural Modelling are two examples (ISM). Unlike the ISM, the ANP is unable to completely reveal all types of interdependence due to its inability to totally rule out the potential of interactions inside the criteria cluster. ISM converts fuzzy, poorly defined mental representations of systems into unambiguous, visible models that can be used for a variety of tasks [142]. The ISM method involves the organization of a variety of elements that are both directly and indirectly related into a thorough, systematic model. The resulting model depicts the structure of a complex problem in a thoroughly thought-out pattern that suggests both images and text [142,143]. There may be several aspects that are connected to a problem for every complex situation under discussion and the situation is described far more correctly by the direct and indirect relationships between the elements than by any one aspect considered separately [143]. ISM therefore builds an understanding of these relationships from a collective perspective. To create the structural mapping of the complex interrelationships between the identified BIM implementation barriers, the study adopted the use of ISM. Fig. 16 depicts the five essential steps.

#### 3.3.1 Defining the elements to be linked

According to the 124 studies that have been compiled, 14 BIMIBs were found. Similar barriers have been combined into one. Table 7 is a summary of the identified barriers and their references.

**Table 7:** Identified BIMIBs in the developing world.

B	List of Barriers	References	T
B1	No/limited awareness and understanding of BIM, it's benefits + ignorance of ROIs + a perception that BIM is not useful at the moment	R1, R2, R4, R5, R6, R7, R10, R13, R14, R21, R23, R24, R26., R27, R28, R30, R32, R37, R39, R40, R43, R45, R46, R49, R52, R53, R54, R57, R58, R62, R63, R64, R66, R67, R69, R71, R72, R74, R76, R77, R81, R84, R87, R88, R89, R92, R93, R95, R96, R99, R100, R101, R102, R103, R104, R107, R111, R112, R114, R116, R117, R119, R120, R121, R122	65
B2	Associated Cost (high cost of software, hardware, etc.)	R2, R4, R5, R7, R9, R10, R11, R12, R13, R15, R16, R17, R21, R23, R24, R25, R27, R28, R29, R33, R34, R36, R37, R38, R39, R40, R42, R43, R45, R46, R51, R53, R55, R56, R57, R58, R59, R60, R64, R65, R66, R67, R70, R71, R73, R75, R78, R80, R81, R82, R88, R89, R90, R92, R93, R94, R97, R99, R102, R103, R107, R109, R111, R112, R113, R114, R115, R118, R119, R120, R121	70
B3	Lack of BIM experience & expertise/skilled personnel	R2, R11, R13, R14, R19, R21, R25, R28, R35, R37, R38, R40, R42, R43, R45, R46, R49, R51, R52, R53, R58, R61, R64, R65, R66, R68, R70, R73, R75, R76, R79, R80, R83, R86, R88, R89, R90, R92, R93, R94, R96, R97, R98, R102, R103, R104, R105, R109, R118, R119, R124	51
B4	Lack of Government support, regulations & incentives	R2, R4, R6, R7, R9, R11, R12, R13, R14, R16, R17, R18, R19, R20, R25, R27, R29, R30, R33, R34, R35, R39, R41, R43, R44, R45, R46, R47, R48, R49, R51, R53 R56, R57, R62, R63, R64, R65, R67, R69, R70, R71, R72, R73, R78, R80, R81, R82, R85, R86, R87, R93, R96, R97, R98, R99, R102, R108, R109, R111, R112, R113, R114, R115, R120, R121	66
B5	Resistance to change + lack of executive buy in and client demand	R2, R4, R6, R7, R9, R10, R11, R13, R14, R15, R16, R17, R18, R20, R21, R23, R24, R25, R28, R33, R34, R37, R38, R39, R40, R42, R43, R44, R46, R47, R49, R50, R51, R52, R53, R56, R58, R61, R62, R63, R67, R68, R69, R70, R73, R75, R76, R77, R80, R81, R87, R89, R90, R92, R93, R94, R96, R97, R99, R100, R101, R102, R105, R106, R107, R111, R114, R115, R116, R117, R124	73
B6	Interoperability & compatibility issues	R2, R3, R5, R6, R7, R9, R11, R19, R23, R25, R31, R33, R41, R45, R55, R62, R65, R67, R70, R73, R76, R86, R90, R114, R118, R122, R123	27
B7	Lack of supporting technology/physical infrastructure/BIM training centers	R4, R5, R6, R7, R9, R10, R15, R20, R21, R23, R24, R28, R29, R30, R33, R34, R39, R40, R41, R42, R45, R46, R47, R49, R51, R53, R57, R58, R59, R61, R68, R69, R70, R72, R80, R81, R83, R89, R90, R92, R94, R100, R101, R103, R105, R109, R112, R115, R118	49
B8	Steep learning curve	R25, R53, R64, R67, R70, R121	6
B9	Unavailability of Contractual & legal framework	R6, R7, R12, R35, R43, R48, R53, R106	8
B10	Data related problems	R6, R7, R9, R15, R18, R19, R33, R39, R41, R43, R44, R45, R65, R77, R85, R86, R93, R104, R106, R107, R108, R118, R121	23
B11	Collaboration & communication issues + unclear roles and responsibilities	R10, R12, R28, R31, R43, R44, R45, R48, R49, R50, R53, R62, R68, R69, R77, R81, R82, R89, R92, R94, R104, R106, R108, R113, R117, R118, R120	27
B12	BIM risks & lack of dispute resolution mechanisms	R12, R48, R100, R101, R111, R113, R114, R115	8
B13	Complex BIM software & tools + BIM process is time consuming & cumbersome	R2, R12, R31, R33, R36, R40, R46, R49, R55, R60, R77, R80, R81, R82, R87, R93, R94, R99, R107, R108, R113, R123, R124	34
B14	Lack of BIM studies in higher educational curricular & the lack of BIM research	R8, R10, R12, R24, R33, R47, R50, R62, R72, R73, R76, R80, R82, R88, R96, R99, R109, R110, R113, R115, R117, R118	16

#### 3.3.2 Creating the correlation structure - structural self-interaction matrix (SSIM)

In this study, the interrelationship between barriers x and y were represented by four symbols: (1) V refers to “barrier x can significantly aggravate barrier y but not vice versa”; (2) A refers to “barrier y can significantly aggravate barrier x but not vice versa”; (3) X refers to “barriers x and y can significantly aggravate each other”; and (4) O refers to “barriers x and y are not related”. Barriers ‘x’ have been represented on the first column of Table 7 whereas barriers ‘y’ are represented on the first row. According to domain knowledge and expert's input, the contextual link between each pair of components was defined, showing whether or not one element leads to another. After being sent the blank version of Table 5 below through LinkedIn, five BIM experts were asked to determine how the BIM barriers interacted with one another by indicating V, A, X or A in the blank cells. These BIM experts had a minimum of 2 years of in BIM usage and industrial experience. The “minority gives way to the majority” approach was used in circumstances where various experts had reached differing conclusions about the interrelationship between the barriers [144]. According to the feedback from the experts, contextual relationships among the fourteen barriers were constructed (see Table 7). Table 8 summarizes the background of experts consulted and their number of years of BIM usage experience.

**Table 8:** The SSIM based on BIM experts’ point of view.

	B1	B2	B3	B4	B5	B6	B7	B8	B9	B10	B11	B12	B13	B14
B1	n/a	V	X	X	X	O	X	V	A	O	A	O	O	X
B2		n/a	A	V	V	O	X	O	O	O	A	V	V	A
B3			n/a	X	X	O	V	V	O	O	A	O	V	X
B4				n/a	A	O	A	A	A	A	A	A	O	A
B5					n/a	V	X	X	V	X	X	V	X	X
B6						n/a	O	A	O	A	A	O	A	A
B7							n/a	A	O	O	A	O	X	X
B8								n/a	O	V	O	O	V	X
B9									n/a	X	A	A	A	O
B10										n/a	X	X	A	A
B11											n/a	V	A	O
B12												n/a	A	O
B13													n/a	A
B14														n/a

#### 3.3.3 Reachability matrix

In a reachability matrix, each cell in the matrix indicates whether the subnet in the row or column can reach the subnet in the corresponding column or row respectively. The SSIM was converted into a binary matrix for further calculation and analysis by substituting the correlation structure's four symbols, V, A, X, and O with the numbers 1 and 0 (see Table 8).

The study's substitution rule, which is illustrated by Table 9, indicates that the (x, y) and (y, x) of the reachability matrix are filled in as the corresponding integers when the symbol for the direction of correlations between two barriers is V, A X, and O [13].

**Table 9:** Initial binary matrix.

	B1	B2	B3	B4	B5	B6	B7	B8	B9	B10	B11	B12	B13	B14
B1	0	0	1	1	1	0	1	0	1	0	1	0	0	1
B2	1	0	1	0	0	0	1	0	0	0	1	0	0	1
B3	1	0	0	1	1	0	0	0	0	0	1	0	0	1
B4	1	1	1	0	1	0	1	1	1	1	1	1	0	1
B5	1	1	1	0	0	0	1	1	0	1	1	0	1	1
B6	0	0	0	0	1	0	0	1	0	1	1	0	1	1
B7	1	1	1	0	1	0	0	1	0	0	1	0	1	1
B8	1	0	1	0	1	0	0	0	0	0	0	0	0	1
B9	0	0	0	0	1	0	0	0	0	1	1	1	1	0
B10	0	0	0	0	1	0	0	1	1	0	1	1	1	1
B11	0	0	0	0	1	0	0	0	0	1	0	0	1	0
B12	0	1	0	0	1	0	0	0	0	1	1	0	1	O
B13	0	1	1	0	1	0	1	1	0	0	0	0	0	1
B14	1	0	1	0	1	0	1	1	0	0	0	0	0	0

The following examples illustrate the substitution rule adopted by the study;

Example 1; (B1, B2) in the SSIM is V, hence, the (B1, B2) in the reachability matrix will be 0, and the (B2, B1) will be 1,

Example 2; (B1, B3) in the SSIM is X, hence, the (B1, B2) in the reachability matrix will be 1, and the (B3, B1) will also be 1,

Example 3; (B1, B9) in the SSIM is A, hence, the (B1, B9) in the reachability matrix will be 1, and the (B9, B1) will be 0,

Example 2; (B1, B6) in the SSIM is O, hence, the (B1, B6) in the reachability matrix will be 0, and the (B3, B1) will also be 0.

In accordance with the aforementioned principle, a preliminary reachability matrix was created to show the interrelationships between each pair of the fourteen barriers. After, in order to determine the final reachability matrix, the ‘SMART Interpretive Structural Model’ was used to calculate the indirect transferability of these barriers after which a power iteration analysis was used to evaluate the transitivity rules [13]. (Find the online version of the ISM software here; http://smartism.sgetm.com/). The transitivity property must be verified in order to acquire the final reachability matrix after obtaining the initial reachability matrix. The rule is, if (x, y) = 1 and (y, z) = 1, then (x, z) = 1 [145]. Incorporating transitivity was represented as 1× by the study. For instance, the relationship between B1 and B6 in Table 10 was represented by 1× which is an indication that there an indirect relationship between B1 and B6 (see Table 11).

**Table 10:** Rule for developing initial reachability.

If the (x, y) Entry in the SSIM Is SSIM	Entry in the initial Reachability matrix (x, y) (y, x)	
V	1	0
A	0	1
X	1	1
O	0	0

**Table 11:** Final + Initial reachability matrix.

	B1	B2	B3	B4	B5	B6	B7	B8	B9	B10	B11	B12	B13	B14	D
B1	1	1	1	1	1	1×	1	1	0	1×	0	1×	1×	1	12
B2	0	1	0	1	1	1×	1	1×	1×	1×	0	1	1	0	10
B3	1	1	1	1	1	1×	1	1	1×	1×	0	1×	1	1	13
B4	1	0	1	1	0	0	0	0	0	0	0	0	0	0	3
B5	1	0	1	1	1	1	1	1	1	1	1	1	1	1	13
B6	0	0	0	1×	1	0	0	0	0	0	0	0	0	0	2
B7	1	1	0	1	1	1×	1	0	1×	1×	0	1×	1	1	11
B8	0	0	0	1	1	1	1	1	1×	1	1×	1×	1	1	11
B9	1	0	0	1	0	1×	0	0	1	1	0	0	0	0	5
B10	1	0	0	1	1	1	0	0	1	1	1	1	0	0	7
B11	1	1	1	1	1	1	1	0	1	1	1	1	0	1×	12
B12	0	0	0	1	0	1×	0	0	1	1	0	1	0	O	5
B13	0	0	0	1×	1	1	1	0	1	1	1	1	1	0	8
B14	1	1	1	1	1	1	1	1	1×	1	0	1×	1	1	13
X	8	6	6	14	10	13	9	6	11	12	5	11	8	7	

1× depicts the indirect relationship between the barriers after transitivity has been tested, D depicts driving power and X depicts dependence power.

#### 3.3.4 Interrelationships and classification of barriers

**Fig. 17:**
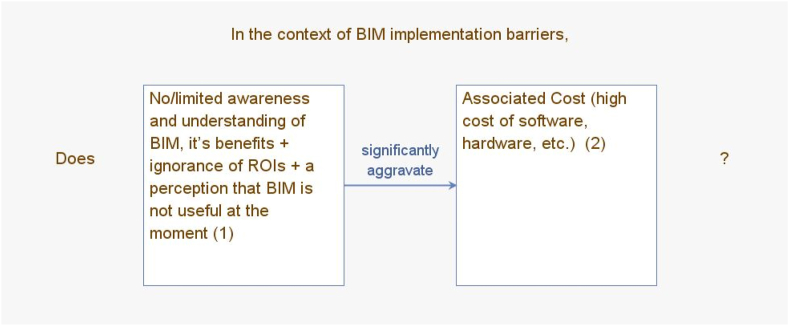
Data entry process for Windows ISM software.

Since the original Interpretive Structural Modelling software Programme was created in 1974 at Battelle Columbus Laboratories, other versions have been generated. In order to partition the different barriers identified into levels and to draw the final ISM model, the study adopted the use of Windows ISM software version created by Drs. Michael Hogan and Benjamin Broome, who are a professor and senior lecturer at the Arizona State University, USA and the NUI, Galway, Ireland respectively. Results from experts on the interrelationships between the barriers were input in the software a pair at a time using the format illustrated in Fig. 17 below. In Fig. 17, for instance, based on the results of the experts' votes, a ‘YES’ or ‘NO’ would be selected from a displayed command on the screen. This process is repeated for all available pairs of barriers. Out of the 14 barriers, there were 91 pairs in total.

**Fig. 18:**
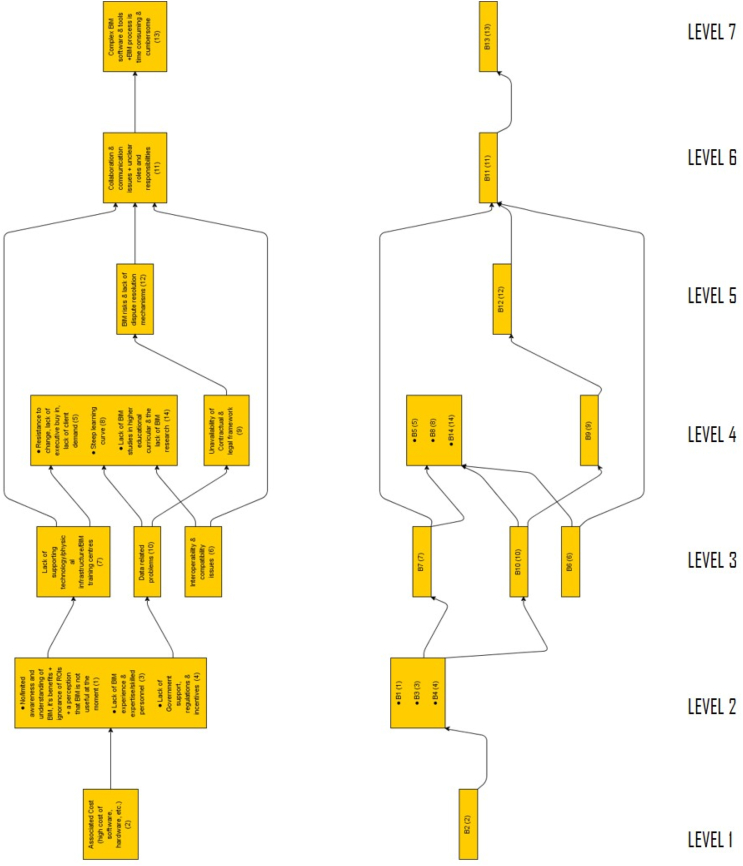
Final ISM model showing the different levels.

Next, a final ISM model that classified the barriers into levels as shown in Fig. 18 was derived. The ISM model showed the connections between the 14 barriers and their hierarchy-level significance ranking from higher to lower. Arrows connect the barriers from bottom (higher level) of the model to the top (lower level). The interconnections of the factors have a significant impact on components that are on top of the model.

**Fig. 19:**
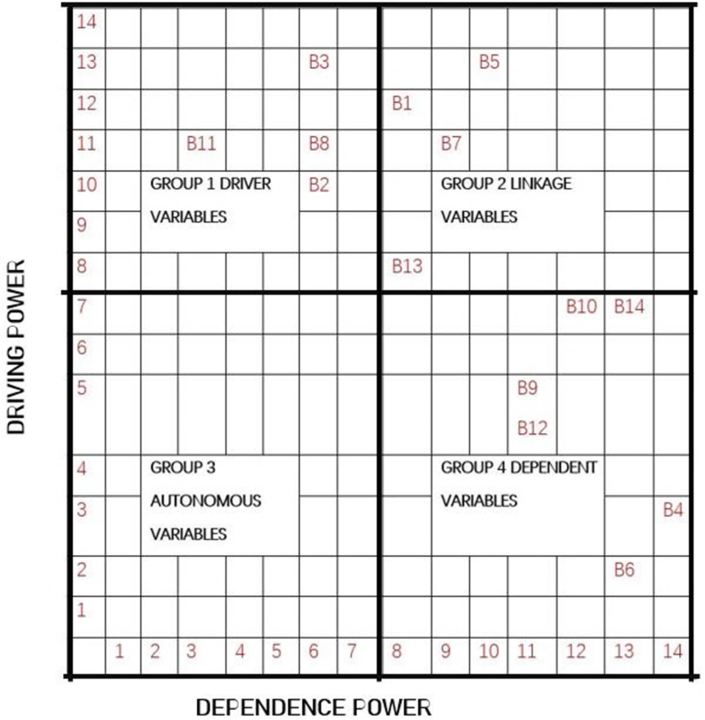
MICMAC analysis.

Lastly, MICMAC ((Matrice d’Impacts Croisés Multiplication Appliquée á un Classement) translated in English as ‘cross-impact matrix multiplication applied to classification’ was used to classify the barriers in order to give a clearer understanding of the interrelationships between the barriers. In MICMAC, variables are mapped onto a two-dimensional grid depending on their values for dependence and driving power, which are shown on the horizontal and vertical axes, respectively. Based on their driving and dependent powers, MICMAC was used to analyze the strength of the relationship between the BIMIBs. Fig. 19 summarizes the results of the MICMAC analysis.

#### 3.3.5 Implications of MICMAC analysis and ISM model results

**Fig. 20:**
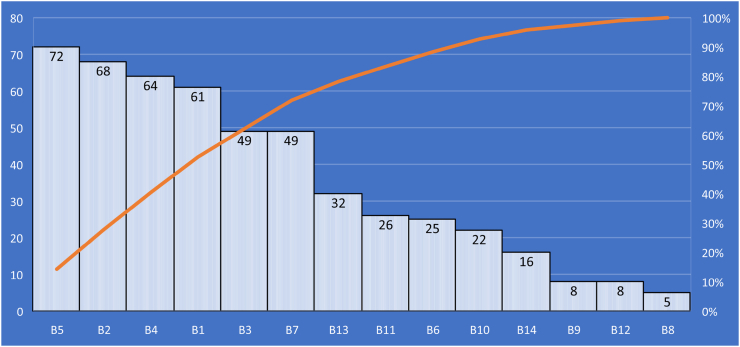
The distribution of the Identified BIM implementation barriers in a descending order of frequency with a cumulative line on a secondary axis as a percentage of the total.

MICMAC analysis groups elements into four clusters based on their driving and dependent power. These groups are: (Group 1) Independent or driver variables: due to the importance of the strong key factors, these independent elements must receive the most attention; (Group 2) Linkage variables: these are connecting factors and are unstable but have the greatest influence on others; (Group 3) Autonomous variables:these variables have no dependence on other factors and are comparatively cut off from the system; (Group 3) Linkage variables; these are predominantly dependent on other factors [145]. Per the MICMAC analysis, B1, B5, B7 and B113 fall under linkage variables. Any effort made to overcome these five barriers will have an impact not just on them but also on other barriers. The barriers B6, B9, B12 and B10 are dependent variables and most likely, they exist as a result of the adverse effects of other barriers. As a result, these barriers do not require urgent attention. The different levels on the ISM model have been discussed below with regards to their position on the MICMAC analysis graph. Some references would be made to the pareto chart in Fig. 20.

##### 3.3.5.1 Level 1

According to the ISM model, the most significant and fundamental barrier that affects BIM implementation in the developing countries is the high associated cost (B2). This can be found at the base of the hierarchy (level 1). Results from the MICMAC analysis indicate that B2 falls under the Independent variables (group 1). Variables in group 1 have the strongest driving power but the weakest dependence power. This is a clear indication that, in an attempt to resolve the BIMIBs in developing countries, there must be great focus on how to deal with the cost associated. Cost is a significant barrier to the implementation of BIM in developing countries, and nearly 90% of the articles studied (see Fig. 20) identified it as such, making it a crucial issue that requires attention. Associated high cost with BIM implementation comes in various forms. There is a high cost involved in the purchase and updating of the BIM software [25,146], a high cost of training BIM experts [147] and a high cost involved in data acquisition and information sharing [148,149]. Investing in BIM technology and starting the process have a disproportionately high initial cost, even when other expenses like training and development are not included. In addition, the intricacy of the BIM technique and the specialized knowledge needed to use it effectively have led to the emergence of a new type of career, the “BIM Expert” whose compensation or remuneration is still seen as a higher-end than that of other normal construction sector positions [150]. Given a greater driving force as illustrated through the MICMAC analysis, eliminating B2 (associated high cost) would make it easier to overcome most of the other barriers.

##### 3.3.5.2 Level 2

After B2, the next significant BIMIBs in the developing world are the high B1(No/limited awareness and understanding of BIM, it's benefits and ROIs), B3(Lack of BIM experience & expertise/skilled personnel) and B4 (lack of governmental support, regulations and incentives). These can be found on level 2. Per the MICMAC analysis, B2 and B3 are also classified under the Independent variables with a very high driving force and but very low dependence power. In Fig. 20, it can be observed that, nearly 90% of the articles studied identified B1, B3 and B4 as part of the top 5 barriers affecting BIM implementation in the developed world. According to the surveys, the majority of stakeholders in the developing countries have little to no understanding of the workings of BIM. Those who have an idea of what BIM are only aware of the BIM terminology, software providers and BIM dimensions. The investigation showed that the understanding of industry professionals on a project's information model requirements, which is one of the key components of implementing BIM, is inadequate. Additionally, there is an excessive mistrust of the software and a lack of knowledge of the BIM capabilities and its development potential [129]. It was discovered in India that enterprises found it difficult to balance the returns on BIM investments, making its adoption a difficult task [86]. The lack of BIM benefit evaluation and BIM supported delivery methods were deemed the most critical of barriers by project owners in the Ethiopian construction industry [151]. In addition, people's aversion to stepping outside of their comfort zones and their unwillingness to begin new workflows contributed to the BIM technology's rejection. The benefits of BIM implementation must always be understood, encompassing both real and intangible benefits; nevertheless, the economic gains given by BIM are frequently vague, which has been considered a major barrier to its acceptance [13]. In addition, 66 out of the 124 studies examined identified the lack of governmental support (B4) as a major BIMIB. A strong support from government is very critical for the successful development and deployment of complex technological systems such as BIM [152]. This is a major barrier in most of the developing countries such as Indonesia [153], India [86], Iraq [68] and Yemen [11]. In Egypt, the study found out that policymakers' involvement in BIM implementation is minimal and they have no discernible influence [112]. Large corporations and educational institutions rather appear to be the key to influencing the adoption of BIM within the Egyptian industry [112]. On the other hand, the study discovered that B4 was not a major barrier in China. This is owing to the fact that the promotion of BIM in China's construction industry has been a national strategy spearheaded by the Chinese government since 2011 [28] and as at 2019, there was a total of 616 construction projects reported to be using the BIM technology in China [154]. It is no wonder that this study discovered earlier in section 3.2.2 that among the developing countries, China is one of the leading countries in BIM implementation. Just as the barriers on level 1, addressing the barriers on this level would go a long way to address most of the other barriers on the subsequent levels.

##### 3.3.5.3 Level 3

After level 2, the next significant BIMIBs can be found on level 3. These are B6 (interoperability and compatibility problems), B7 (lack of supporting technology and infrastructure for the BIM software and process) and B10 (data related problems). Barriers on this level can be classified as technical and technological barriers. Interoperability, which is the ability of various software types to communicate with one another, has gained popularity in the AEC sector [155]. Making BIM truly interoperable would require a lot of work because there are hundreds of different file formats, each of which was developed for a specific purpose [155]. About 35% of the studies identified interoperability as a major barrier. There is also the problem of the lack of compatibility of the BIM standards with local standards. Since the software is produced outside of the country, it does not take its cues from regional standards' quirks, so nearly all of its nomenclature needs to be changed just because they do not adhere to regional specifications [133]. With regards to the data related barriers, where cities are smartly connected with strong IoT power, the threat of location-based information being acquired by BD applications and transferred via networks results in a heightened risk of privacy [156]. As a result, the threat to data privacy and security is becoming increasingly prevalent as additional data sources become pervasive. in addition, extremely sensitive data exchanged over IoT without clear ownership leads to a slew of errors and, in certain cases, inconsistency, which can lead to misinterpretations. In addition, Stakeholders may face new risks as a result of BIM implementation. The veracity of the information provided in BIM, for example, implies significant risks. Inaccuracies lead to poor decision-making and more time and money spent correcting the errors that ensue. As a result, it is required to design and secure insurance that is suitable for BIM deployment. In order to limit potential risks, determining who is accountable for checking the information in BIM and what should be done if inaccuracies are discovered in the model should be common practice [13]. This is because there is no insurance in place to deal with faults and flaws in a BIM, a lot of time and effort is spent requesting and augmenting incomplete and wrong data.

##### 3.3.5.4 Level 4

**Fig. 21:**
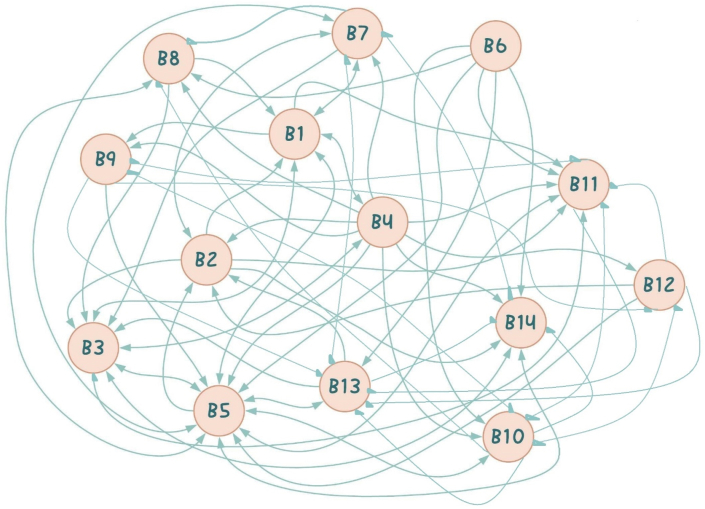
Summaries everything that has been discussed so far, that is, the interrelationships between all 14 barriers.

Barriers on level 4 include B14 (the lack of BIM studies in the educational curricular of higher institutions and the general lack of BIM research), B5 (general resistance or unwillingness on the part of stakeholders to change), B8 (a steep learning curve) and B9 (the unavailability of Contractual & legal framework). 73 out of the 124 studies reviewed identified B5 as a major BIMIB, making it the most occurring barrier in the developing world. The position of B5 on the ISM model is a reflection of the fact that this barrier exists as a result of other barriers. A careful look at Fig. 21 reveals so many arrows leading to B5. This means that B5 exists as a result of barriers such as B2 (high associated cost), B1(No/limited awareness and understanding of BIM, it's benefits and ROIs), B3(Lack of BIM experience & expertise/skilled personnel, B4 (lack of governmental support, regulations and incentives), B6 (interoperability and compatibility problems), B7 (lack of supporting technology and infrastructure for the BIM software and process) and B10 (data related problems). In addition, according to the MICMAC analysis, B5 is also considered a linkage variable, hence, resolving it would result in resolving other barriers.

##### 3.3.5.5 Levels 5, 6 and 7

These levels are made up of B12 (lack of dispute resolution mechanisms and the fear of BIM risks), B11 (the lack of collaboration and the existences of communication issues) and B13 (complex BIM software and tools) respectively. These barriers being the last on the hierarchy is an indication that there is not much to worry about them if all the previous barriers are well sorted out. They exist as a result of the existence of other barriers. The study found out that BIM practice in the developing world is disjointed and non-collaborative. As a result, the majority of BIM users utilize the technology for stand-alone functions like visualizing during 2D design processes and creating conventional 2D technical documentation [130]. Cloud based BIM models are entirely non-existent. The majority of AEC firms that use BIM operate autonomously, and each firm has its own [130]. The ideal scenario for BIM deployment is for a group of people to collaborate on a single integrated BIM model, such as the client, designer, engineer, and contractor. However, the possibility for such integration is restricted, and prior research have found that stakeholders have negative attitudes regarding cooperating [13]. This barrier coupled with the problem of unclear roles and responsibilities are a major BIMIB in the developing world.

Generally, the summary is that, according to the ISM model, in order to curb BIMIBs in the developing world, a lot of attention needs to be paid to the barriers on levels 1 and 2, which are B1, B2, B3 and B4. Addressing these barriers would directly and indirectly address all other barriers.

In Fig. 21, an arrow pointing from one barrier(x) to another(y) is an indication that barrier(x) significantly aggravates barrier(y) and a two-way arrow is an indication of a mutual relationship.

## 4 Recommendations for BIM implementation in the developing countries

**Fig. 22:**
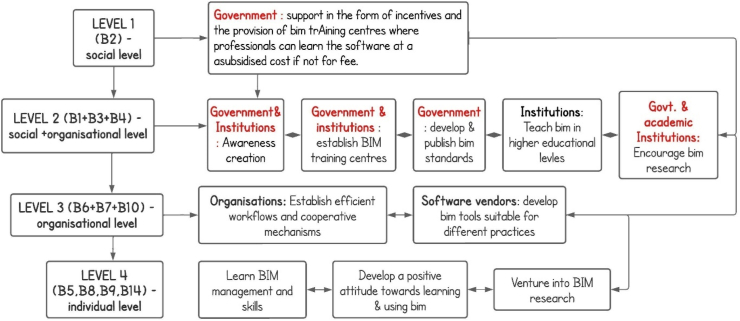
How we came about the recommended 3-level strategic plan.

Based on the ISM model, the interrelationships between the barriers and the MICMAC analysis, the study proposes a 3 - level plan to help address the BIMIBs in the developing world; (1) the social level, (2) organizational level and (3) the individual level. The social level deals with the role of government in solving the problem. The organizational level has to with the role of organizations such as tertiary institutions, firms in AEC industries, software developing companies, manufactures, etc. Lastly, individual level has to do with the role of stakeholders such as clients, architects, engineers, academicians, etc. In the AEC industry. Fig. 22 summarizes the recommendations.

Based on the findings of this study, in tackling the BIMIBs in the developing countries, government's role appears at least six (6) times out of 11 strategies proposed by the study. Government intervention is needed to solve majority of the problems at level 1 & 2 which when resolved, would tackle most of the other barriers on the subsequent levels. Our conclusion is that majority of the problems that exist in the developing countries are largely because of the lack of governmental support for BIM implementation. This assumption is supported by findings from several studies which have shown that even though BIM research has advanced greatly, the governance component of implementing BIM, creating frameworks, and advancing the research in the developing world has not been extensively tapped [6]. Government intervention is needed in subsidizing the costs associated with the BIM software and its implementation (B2), very crucial in the provision of supporting infrastructure (B7) and in the creation of BIM training centers for professionals (B3). Government intervention is crucial in the development of BIM standards (B4) and partly crucial in the promotion of research on BIM and its inclusion in the academic curricular (B14) of higher education institutions. Mandatory BIM standards directly and indirectly affect the choices of clients and firms when it comes to adopting the use BIM in projects (B5). Government intervention is required in sensitizing the people and in the creation of awareness on BIM and its benefits (B1). In order to control the adoption of BIM in the architectural, engineering, and construction sectors of any nation, government's proactive action is required. Governments around the world can show their support by promoting national strategies that support BIM implementation, such as investing in collaborative research and development relationships between universities and industries, lowering tax rates on innovation and intellectual property, developing national standards for BIM implementation, and funding BIM training schemes [152]. The USA for instance, is currently the largest producer and consumer of BIM products and solutions and one of the BIM technology's pioneering nations which is not surprising because in the USA, for the purpose of promoting and developing BIM applications, numerous government and non-government organizations work together concurrently [157]. The UK is now a global leader in BIM largely due to the implementation of the government's policy, making room for the promotion of British standards abroad leading to an increase in construction exports [158]. Even though this study proposes a 3-level strategy for addressing the barriers, the study would recommend a strong focus on the role of Government in BIM implementation in the developing world. The study would therefore recommend a further research to discover the root causes of the lack of governmental support for BIM implementation in developing countries and to provide a workable framework that can serve as a benchmark for alleviating this problem.

## 5 Conclusion

Through a combination of bibliometric review, interpretive structural modelling and MICMAC analysis, the study identified and classified the top 14 barriers to successful BIM implementation in developing nations based on the interrelationships between these barriers. This was achieved through a critical examination of peer-reviewed literature primarily based on empirical investigations and the knowledgeable input of 5 BIM experts. With innovative disruptions from related sectors within the construction industry, BIM research has attracted global interest. The reviewed publications, which come from several developing countries, were exported from the Web of Science Core Collection of Indexed Research Documents. In general, developing countries continue to have difficulty adopting BIM. However, the current state of affairs is positive given that there is evidence that researchers are producing more research in this field as the days go by.

The study discovered, among other things, that researchers didn't work together. Instead, they tended to operate independently. The study recommends a concerted and cooperative effort from scholars. Additionally, the study found that the most fundamental of all of the BIMIBs in the developing world is associated high cost, followed by the lack of governmental support, lack or expertise and unawareness or ignorance of what BIM is and its benefits. From the 3-level strategical framework proposed by the study to help mitigate these barriers, the study established government's role and involvement as the topmost and most sustainable strategy.

The current state of the BIMIB research is visualized in this study using visualization technologies, which offers useful information for researchers and practitioners. With the development of the current study, important academics, places, and articles were recognized. This piece lays forth the knowledge base for next research. The study has a limitationdespite its extensive examination and numerous major contributions to the corpus of knowledge. This study only included documents from the Web of Science Core Collection of Indexed Articles. Future research should combine information from a variety of databases.

## Author contribution statement

All authors listed have significantly contributed to the development and the writing of this article.

## Data availability statement

Data will be made available on request.

## Funding

This study was supported by research on part and components library and building information model sub part, coding and labelling standards (2022-K-069). The study was also supported by National Key R&D Program of China - Strategic Scientific and Technological Innovation Cooperation Joint Research and Demonstration of Green Low-carbon Renovation of Existing Buildings and the Technology of Carbon Neutralization (2022YFE0208600）

## Declaration of competing interest

The authors declare that they have no known competing financial interests or personal relationships that could have appeared to influence the work reported in this paper.
